# Supplementary material for: Cathelicidin Attenuates Hyperoxia-Induced Lung Injury by Inhibiting Ferroptosis in Newborn Rats
Source: Antioxidants (Basel). 2022 Dec 4;11(12):2405. doi: 10.3390/antiox11122405 (PMC9774284; doi:10.3390/antiox11122405)
Supplement: Supplementary file 1 [file antioxidants-11-02405-s001.zip › antioxidants-1986476-supplementary.pdf]

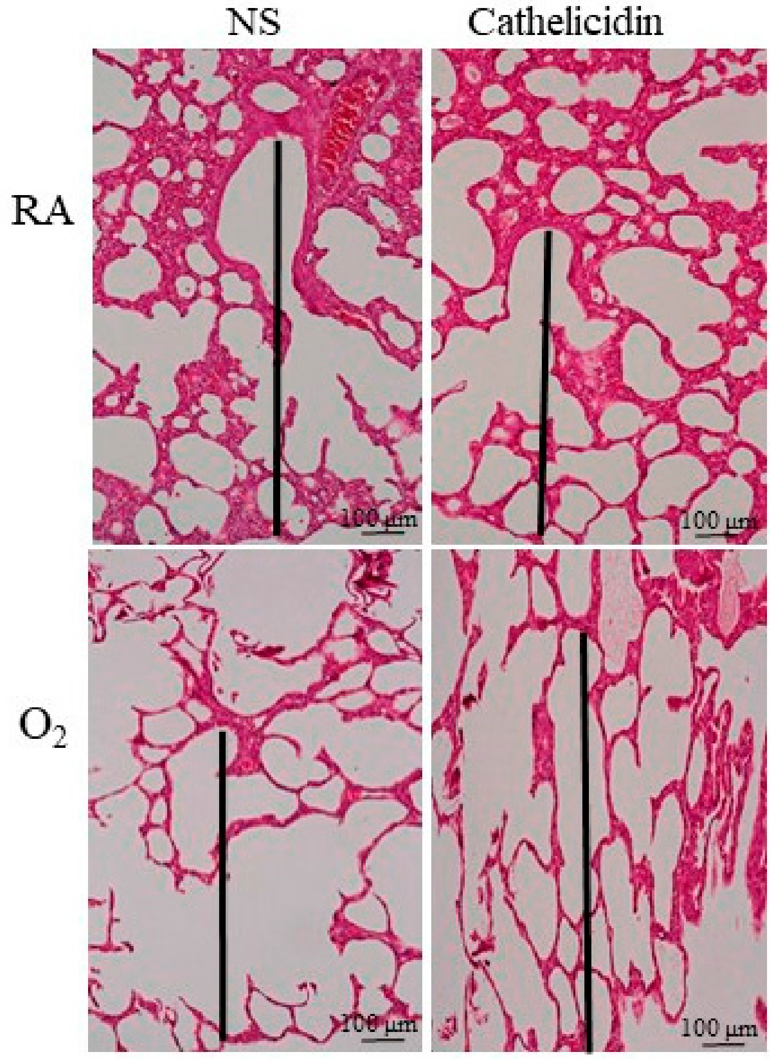

Supplementary Figure S1. RAC was measured by counting the number of alveoli passed by a vertical line from the center of the respiratory bronchioles to the border of the pleura.
